# Occult hepatitis B virus infection: influence of S protein variants

**DOI:** 10.1186/s12985-016-0464-z

**Published:** 2016-01-19

**Authors:** Zhenhua Zhang, Ling Zhang, Yu Dai, Yafei Zhang, Jun Li, Xu Li

**Affiliations:** Department of Infectious Diseases, the First Affiliated Hospital, Anhui Medical University, Hefei, 230022 China; School of Pharmacy, Anhui Medical University, Hefei, China

**Keywords:** Hepatitis B surface antigen, Hepatitis B virus, Occult, Variant

## Abstract

**Background:**

In occult hepatitis B viral infection (OBI), the persistence of hepatitis B virus (HBV) DNA is associated with a lack of hepatitis B surface antigen (HBsAg). To assess the possible role of HBsAg immune escape variants in OBI patients, variability in the HBV S gene was evaluated for OBI patients as well as chronic HBV infection patients from the same families.

**Methods:**

We selected 17 HBV DNA-positive/HBsAg-negative patients (OBI group) and 15 HBV DNA- and HBsAg-positive patients from OBI families (control group). The S gene was amplified and cloned, and at least 15 clones per patient were sequenced and analyzed.

**Results:**

Although the incidence of stop codon mutations within the S region was higher in the OBI group (13.6 %) than in the control group (1.5 %, *P* < 0.001), this type of mutation, together with insertion and deletion mutations, was prevalent in only three OBI patients. In the major hydrophilic region (MHR), a median of 0.75 residues were altered in every 100 residues for the OBI patients, whereas 0.95 out of 100 residues were changed in the control group (*P* = 0.428). Furthermore, some variants that are generally considered immune escape variants, such as mutations at positions s145, s147, and s123, were only observed in less than 5 % of all the clones sequenced, in either OBI or control group.

**Conclusions:**

Our data suggest that HBsAg variants may not play a major role in OBI pathogenesis.

## Background

More than 240 million people are chronic carriers of the hepatitis B virus (HBV) worldwide, posing a serious public health problem [1, 2]. In the natural course of HBV infection, virus clearance is classically characterized by the loss of hepatitis B surface antigen (HBsAg) and HBV DNA in the serological profile of the patient. Notably, several reports have described the persistence of HBV DNA in 0–76.2 % of HBsAg-negative patients [3–7]. Diagnostically, this phenomenon, named occult hepatitis B infection (OBI), is defined as the presence of HBV DNA in the patient’s serum and/or hepatocytes, but a lack of serum HBsAg [8]. OBI is considered a high potential risk factor for hepatitis, cirrhosis, post transfusion hepatitis (PTH), and hepatocellular carcinoma (HCC) [3, 8–11]. While clinical investigation is ongoing, the mechanisms underlying the presence of HBV DNA and lack of HBsAg during OBI are not completely understood.

Previous work has implied that OBI development is possibly related to mutations in the S gene of the virus, which result in the production of escape mutants that go undetected by the host’s immune system [12–14]. The HBV S gene (or envelope gene) has three open reading frames (ORFs), which form three distinct mRNA moieties that encode the small, medium, and large HBsAgs. HBsAg mutations at single or multiple sites in the “α” determinant of the S protein may emerge as a result of selective pressure. This “α” dominant epitope of the HBsAg is located from amino acid 124 to 147 within the major hydrophilic region (MHR). Some case reports have indicated that there are a relatively large number of amino acid changes in this section of the HBsAg in OBI patients [12–18]. Moreover, site-directed mutagenesis experiments, in which an amino acid was replaced with all other possible residues, have confirmed that some amino acid mutations in the “α” determinant, including G145R, D144A, P142S, Q129H, and M133L, could lead to decreased HBsAg binding and HBsAg test failure [18–21]. However, no specific S gene mutations previously studied have been clinically detected in a large enough number of OBI cases to indicate causation [22–26]. We believe that the merit of these former studies is limited by the small number of clones or patients analyzed and the fact that the prevalence of naturally occurring mutations in the “α” determinant is unknown, particularly in OBI patients.

In this study, we assessed the role of HBsAg immune escape variant selection in OBI by evaluating the level of variation present in the HBV S gene in a large number of OBI patients as well as patients with chronic HBV from the same families.

## Results

### Clustering of 15 OBI-affected families

A total of 747 HBsAg-negative sera samples were collected from 265 families in which at least one family member had a chronic HBV infection. Of these samples, 60 (8.0 %) were identified as OBI. In order to explore the mechanisms underlying OBI, we investigated 17 of the patients diagnosed with OBI from 15 families (F1–F15) which also include a member with chronic HBV infection.

**Fig. 1:**
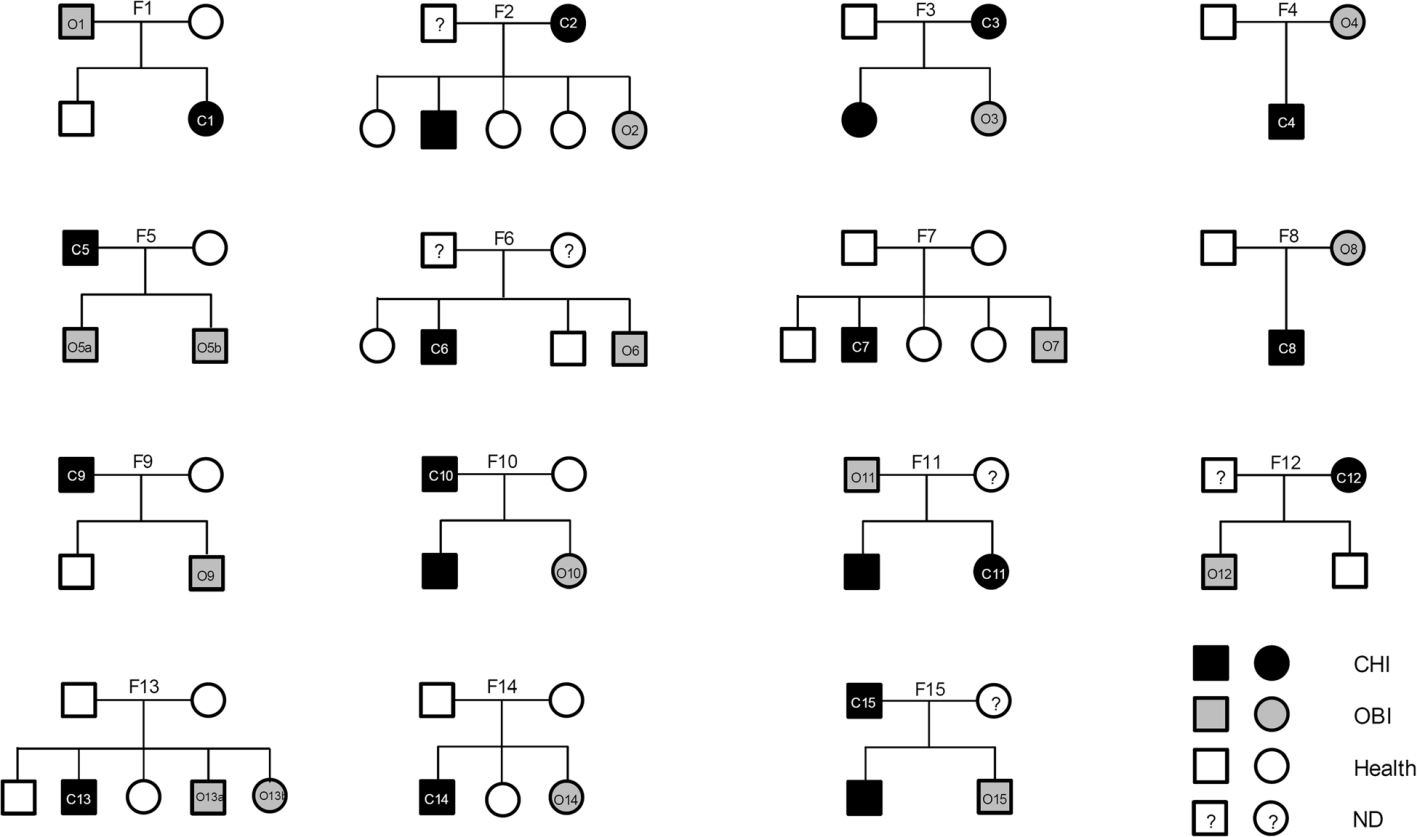
Distribution of OBI and control patients in 15 families included in this study. Symbols: square, male; circle, female; O, OBI patient; C, control patient; CHI, chronic HBV infection patient; ND, not detected. Each family was assigned an ID number. If a family had two OBI or control patients, a, b were used to specify them respectively

Due to death of a family member and other unforeseen events, we were unable to obtain serum samples from some members of these 15 families. Of the samples collected from these families, 44 individuals were negative for HBsAg, among which 17 were diagnosed as OBI. Only 5 of these families (F2, F3, F10, F11, and F15) had 2 members with chronic HBV infection, whereas 2 families had 2 OBI patients. The relationship between a patient with chronic HBV infection and a patient with OBI could be either parent/child or sibling (Fig. 1). Notably, most of the OBI patients did not manifest any clinical symptoms, with the exception of one patient who presented with fatigue, loss of appetite, and elevated alanine aminotransferase (ALT) in multiple laboratory examinations. This patient was free of human immunodeficiency virus (HIV) and hepatitis C virus (HCV) infections, adiposis hepatica, drug-induced hepatitis, and autoimmune hepatitis, and refused to accept experimental anti-viral therapy. Among patients in the control group, 10 were diagnosed with chronic HBV infection, whereas 3 were diagnosed with cirrhosis and 2 appeared to be asymptomatic HBV carriers (Table 1). Notably, the OBI and control groups were comparable with regard to gender bias (10 male patients were present in each group, *P* = 0.647) and median age (34 years in both groups, *P* = 0.395) (Table 1). All patients were negative for HCV and HIV antibodies.

**Table 1:** Main patient characteristics

Family	Patient	Sex	Age	Diagnosis	Kinship	Serological markers					Viral load	ALT	Genotype
			years			HBsAg	Anti-HBs	HBeAg	Anti-HBe	Anti-HBc	(log 10 copies/ml)	U/L	(Major/Minor)
F1	C1	F	30	CHB	-	+	<10	-	+	+	<3.0	47	C/B
	O1	M	57	OBI	Father	-	175	-	-	-	<3.0	37	C
F2	C2	F	69	LC	-	+	<10	-	+	+	5.9	36	C
	O2	F	39	OBI	Daughter	-	31	-	-	+	<3.0	15	C
F3	C3	F	52	CHB	-	+	<10	+	-	+	3.1	76	C
	O3	M	27	OBI	Son	-	>1000	-	-	-	<3.0	27	B/C
F4	C4	M	19	CHB	-	+	<10	-	+	+	<3.0	143	C
	O4	F	49	OBI	Mother	-	59	-	-	-	<3.0	16	C
F5	C5	M	32	ASC	-	+	<10	-	-	+	<3.0	34	C
	O5a	F	10	OBI	Daughter	-	<10	-	-	-	<3.0	11	C
	O5b	M	5	OBI	Son	-	49	-	-	-	<3.0	10	C
F6	C6	M	48	CHB	-	+	<10	+	-	+	4.1	75	B/C
	O6	M	46	OBI	Brother	-	<10	-	+	+	<3.0	16	C
F7	C7	M	31	CHB	-	+	<10	+	-	+	5.3	54	C
	O7	M	29	OBI	Brother	-	52	-	-	+	<3.0	9	C
F8	C8	M	19	CHB	-	+	<10	-	+	+	<3.0	85	C
	O8	F	44	OBI	Mother	-	<10	-	-	-	<3.0	27	C/B
F9	C9	M	36	CHB	-	+	<10	+	-	+	5.7	105	C
	O9	M	9	OBI	Son	-	>1000	-	-	-	<3.0	13	C
F10	C10	M	56	LC	-	+	<10	-	+	+	<3.0	68	C
	O10	F	19	OBI	Daughter	-	<10	-	-	+	<3.0	194	C
F11	C11	F	22	CHB	-	+	<10	-	-	+	5.1	59	C
	O11	M	44	OBI	Father	-	56	-	-	-	<3.0	19	C
F12	C12	F	34	ASC	-	+	<10	-	-	+	4.0	15	C
	O12	M	14	OBI	Son	-	255	-	-	-	<3.0	15	C
F13	C13	M	35	CHB	-	+	<10	+	-	+	<3.0	1678	B/C
	O13a	F	40	OBI	Sister	-	<10	-	-	-	<3.0	25	B/C
	O13b	M	34	OBI	Brother	-	<10	-	-	-	<3.0	18	B/C
F14	C14	M	18	CHB	-	+	<10	+	-	+	6.3	316	C
	O14	F	23	OBI	Sister	-	39	-	-	+	<3.0	9	C/B
F15	C15	M	59	LC	-	+	<10	-	-	+	<3.0	59	C
	O15	M	36	OBI	Son	-	132	-	-	+	<3.0	34	C

*C* control, *O* occult hepatitis B infection, *F* female, *M* male, ASC asymptomatic HBV carrier, *CHB* chronic hepatitis B, *LC* liver cirrhosis, *ALT* Alanine aminotransferase, O5a and O5b come from one family, O13a and O13b come from one family

### Virological characteristics

As shown in Table 1, HBV genotype distributions were heterogeneous in both groups, and genotype B/C co-infection was also observed (5 and 3 patients in the OBI group and control group, respectively, *P* = 0.539). In fact, 12 patients with OBI (70.6 %) had the same genotype as their family members with chronic HBV infection. Among the other 5 OBI patients, the majority of clones (17, 18, 15 out of 19, 20, and 19 clones) were also observed to be genotypically similar to their family members with chronic HBV infection. In the OBI group, all patients were negative for HBsAg and HBV e antigen (HBeAg). At the same time, the HBV DNA titers were less than 1000 copies/ml in all patients, whereas the levels of HBsAg-specific antibodies (anti-HBs) in patients with OBI were rather high, with 11 patients having an anti-HB titer greater than 10 IU/l and a median titer of 49 mIU/ml. In contrast, the frequency of OBI patients positive for HBeAg- and HBV core-specific antibodies (anti-HBe, 1 in 17 and anti-HBc, 6 in 17) was lower than that found in the control group (5 in 15, *P* = 0.047 and 15 in 15, *P* < 0.001, respectively). The patients in the control group also had a higher viral load, with a median of 3.1 log10 copies/ml (range, < 3 to 6.1, *P* < 0.001), as well as higher ALT levels (median: 16 and 68 U/L for the OBI and control groups, respectively, *P* < 0.001).

### Surface region mutations

A cloning-sequencing approach was used to study the entire S gene sequence for patients from each group. In order to standardize our comparison, we selected two standard reference sequences: genotype B (GQ205440) and genotype C (GQ205441), both of which are found in the Chinese population [27].

**Fig. 2:**
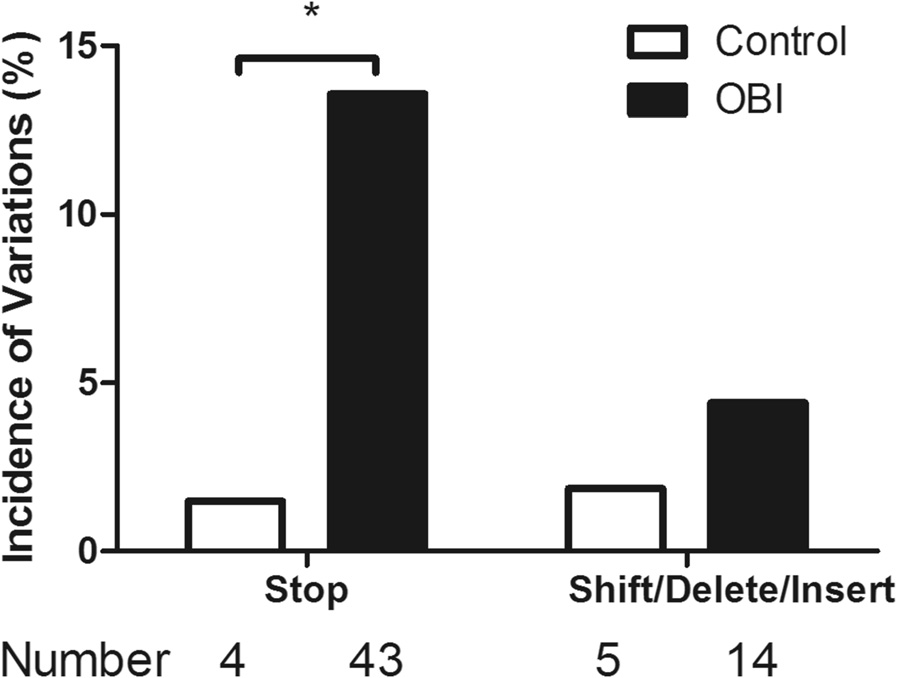
Frequencies of clones with stop codon, insertion, deletion and reading frame shift mutations in the full-length S gene for OBI group (*black bars*) and Control group (*white bars*). Statistical significance was calculated by using the ANOVA test. *, *P* < 0.05

Clones from 10 patients in the OBI group and 5 patients in the control group harbored termination, insertion, or deletion (TID) mutations in the HBV S gene. Amino acids variations in the OBI group and the control group were compared (Table 2, Fig. 2). Among these mutations, some nucleotide insertions or deletions caused reading frame shifts in the S protein of OBI patients (5 clones) as well as in the control group (1 clone). The frequency of stop codon mutations in the OBI group (13.6 %) was significantly higher than that found in the control group (1.5 %, *P* < 0.001). Further, the incidence of insertion or deletion mutations in patients in the OBI group (4.4 %) was also higher than that in the control group (1.9 %), although this difference was not statistically significant (*P* = 0.084). Notably, 3 patients with OBI had TID mutations in more than 50 % of the clones that contained sequences encoding the S protein. In these patients, the antigenicity of HBsAg was likely to be altered, potentially losing its ability to induce antibody production entirely. Interestingly, the presence of mutated clones always appeared to be associated with the detection of at least one wild-type HBV clone in each patient, and the same patient could be infected with a mixture of HBV strains incorporating a variety of residue changes in the S protein.

**Table 2:** Distribution of stop codon, insertion, deletion and reading frame shift mutations in the full-length S protein

OBI group							Control group						
Patient	Genotype	N^a^	Mutation^b^	Type	Site (aa)	Note^c^	Patient	Genotype	N	Mutation^a^	Type	Site (aa)	Note^b^
O1	C	3	TGT → TGA	Stop	69		C1	B	1	TTG → TAG	Stop	209	
	C	4	TGG → TGA	Stop	182								
	C	3	AGCACGGGACCA	Deletion	117-120	STGP							
O2	C	1	TTT	Deletion	220	F							
O3	B	1	TGG → TAG	Stop	35								
O5a	C	2	TGG → TAG	Stop	163								
	C	1	Add “T” nt638	Shift	213								
O6	C	1	TTA → TGA	Stop	217		C6	B	1	Miss “G” nt606	Shift	203	
O8	B	1	Miss “G” nt54	Shift	20								
	C	1	Add “T” nt607	Shift	203								
	C	1	Miss “G” nt54	Shift	20								
	C	1	Miss “T” nt240	Shift	80								
O9	C	2	TTA → TAA	Stop	216								
	C	4	ACC	Insert	124-125	T							
							C10	C	3	AACAACAAC	Insert	116-117	TTT
								C	2	TGG → TAG	Stop	195	
O11	C	8	TTA → TAA	Stop	216		C11	C	1	AACAACAAC	Insert	116-117	TTT
	C	1	AACAACAAC	Insert	117-118	TTT							
O13a	B	8	TGT → TGA	Stop	107		C13	B	1	CAG → TAG	Stop	30	
	C	7	TTA → TAA	Stop	216								
O15	C	6	TTA → TAA	Stop	216								

*Shift* reading frame shift, *Insert* insertional mutation, *Deletion* deletion mutation, *Stop* stop codon mutation

^a^ the number of clones with stop codon, insertion, deletion or reading frame shift mutations in the full-length S protein

^b^ the different types of nucleotide variation, e.g. TGT → TGA indicate the nucleotide sequence change into TGA from TGT; “AGCACGGGACCA” et al. is the nucleotide sequence of insertions or deletions; Add “T” nt638 indicate to add a base”T” after nt638 of S gene; Miss “G” nt54 indicate to miss a base”G” after nt54 of S gene

^c^ the amino acid sequence of insertions or deletions

^b, c^ Reference sequence: genotype B (GQ205440), genotype C (GQ205441)

Although TID and frameshift mutations appear to occur in both OBI and control patients in each family, the site and type of the mutations varied for each individual patient. TID mutations were less frequently found in clones from patients in the control group. For instance, TID mutants were found in only 1 clone from control patients from families 1, 6, 11, and 13. Furthermore, in family 10, no TID mutant was found in the OBI patient, but insertion or termination mutations existed in 5 clones (26.3 %) from the control patients. In the families with 2 OBI patients (F5, F13), TID mutations were found in only 1 of the OBI patients (O5a, O13a), but not the other (O5b, O13b), and these mutations were not observed in the control patient (C5, C13) in either family (Table 2).

### Statistical analysis of mutations occurring in the OBI and control groups

**Fig. 3:**
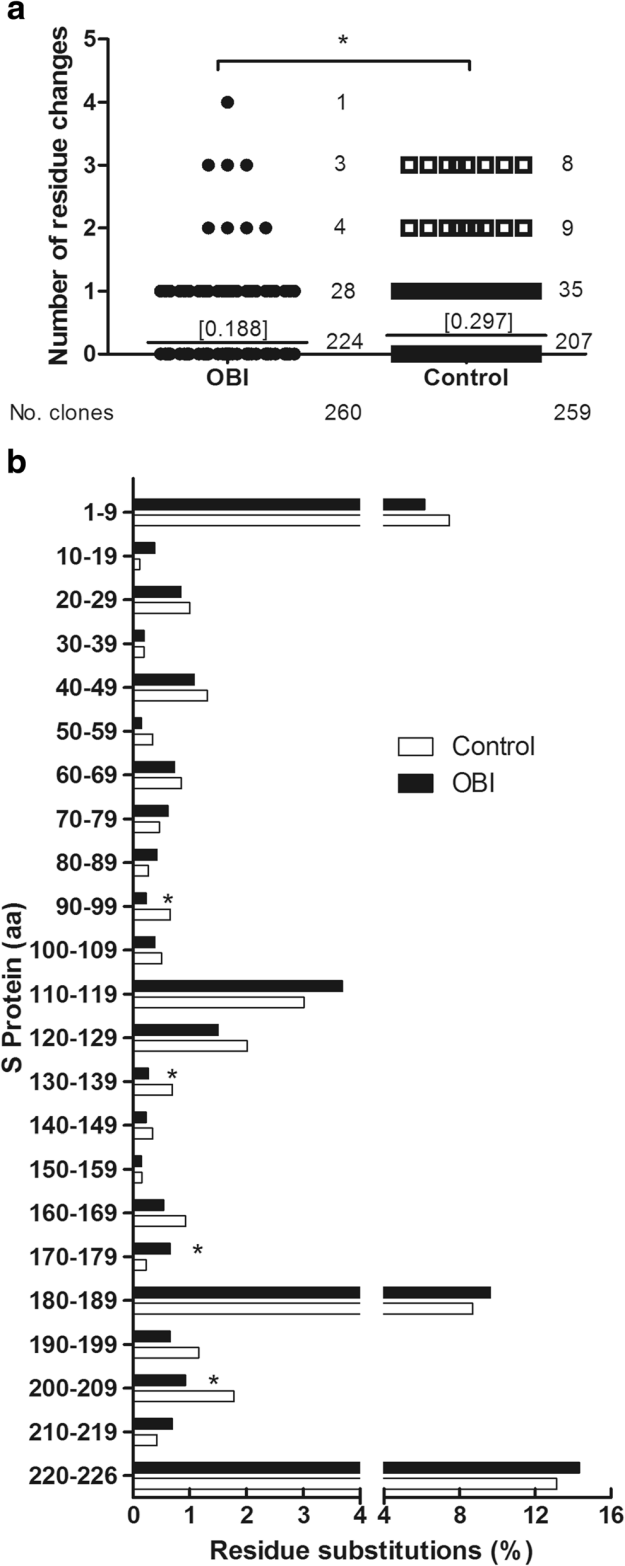
**a**. Numbers of residue changes in clones from OBI group and Control group patients within the “α” determinant of the MHR. The numbers for the 260 clones (*circles*) in OBI group and the 259 clones (*squares*) in Control group are distributed according to the numbers of residue changes in the “α” determinant. Lines and numbers above them in parentheses, means of amino acid mutation/clone in the “α” determinant; numbers on the right side of circles or squares, numbers of clones. Statistical significance was calculated using a Mann–Whitney test. **b**. Frequencies of residue substitutions within the S protein in isolated clones from HBsAg- negative patients (OBI group, black bars, *n* = 17) and HBsAg- positive patients (Control group, white bars, *n* = 15), analyzed in intervals of 10 amino acids each. Each bar represents the percentage of mutated residues for all clones at each interval of 10 amino acids per group. *, *P* < 0.05

A comparison of all clone sequences (excluding clones that incorporated TID mutations) from each patient was performed. The clone sequences derived from individual patients were then compared to a consensus sequence of the same genotype to identify uncommon residues. The percentages of amino acid residue substitutions were also compared between the OBI group and the control group. In the 17 OBI patients, most clones were highly homologous to those from their HBV-infected family members. The 226 amino acids of the S protein can be divided into three regions: the N-terminal region (1 to 99), the MHR (100 to 169; which includes the “α” determinant from 124 to 147), and the C-terminal region (170 to 226). Our analysis indicates that the distribution of amino acid substitutions in the S protein was heterogeneous, and the frequency/accumulation of these changes in most areas of the protein was similar between the two groups (*P* = 0.461) (Table 3). In all patients, the C-terminal region of the protein appeared to be more variable in terms of residue substitution (3.95/100 amino acids in the OBI group and 3.79/100 amino acids in the control group) compared to the N-terminal region or the MHR (Table 3). In the MHR, a median of 0.75 residues were altered in every 100 residues for the OBI patients, and 0.95 out of 100 residues were changed in the control group (*P* = 0.428). Notably, although multiple mutations were observed within the “α” determinant in both groups (36 clones, 13.8 % in the OBI group and 52 clones, 20.1 % in the control group), the residue changes in OBI group patients within the “α” determinant was less frequent than that in control group patients (averaging 0.188 and 0.297 amino acid mutations per clone, respectively, *P* = 0.049) (Fig. 3a). However, the mutation frequency in this site was not significantly different between the OBI group (0.69 mutated residues per 100 amino acids) and the control group (1.16 mutated residues per 100 amino acids, *P* = 0.307) (Table 3). Thus, we doubt that this difference in the accumulation of mutated residues in the “α” determinant between the OBI and HBV groups would be enough to significantly alter the immunogenicity of the S protein.

**Table 3:** Distribution of amino acid changes in the full-length S protein and its different regions

OBI group	No. of residue changes/100 aa^a^					Control group	No. of residue changes/100 aa^a^				
	aa 1–226	aa 1–99	aa 100–169	aa 124–147	aa 170–226		aa 1–226	aa 1–99	aa 100–169	aa 124–147	aa 170–226
O1	2.41	2.24	0.79	1.85	4.68	C1	1.70	1.12	0.83	1.54	3.79
O2	1.37	1.01	0.75	0.44	2.77	C2	1.71	0.38	0.89	0.00	5.04
O3	1.43	1.07	0.59	0.74	3.10	C3	1.51	1.17	0.23	0.00	3.69
O4	1.92	1.57	0.79	0.69	3.90	C4	1.86	1.48	0.86	0.56	3.74
O5a	2.16	1.90	1.60	1.47	3.30	C5	1.11	0.90	0.24	0.23	2.53
O5b	1.42	1.01	0.14	0.00	3.68						
O6	2.16	1.31	1.09	1.23	4.95	C6	2.47	2.02	2.86	4.17	2.77
O7	1.13	0.39	0.56	0.00	3.12	C7	2.26	2.50	1.18	2.45	3.20
O8	1.49	1.08	0.31	0.00	3.63	C8	2.29	2.13	1.19	1.16	3.90
O9	1.73	0.81	0.57	0.00	4.74	C9	1.99	0.86	2.00	2.29	3.95
O10	1.93	0.48	1.68	0.74	4.75	C10	1.77	1.01	1.02	1.49	4.01
O11	1.37	0.40	1.29	2.08	3.16	C11	1.96	0.38	1.70	1.30	5.04
O12	1.43	0.56	0.56	0.00	4.00	C12	1.62	0.67	1.81	2.22	3.04
O13a	2.21	1.11	1.64	1.04	4.82	C13	1.23	0.79	0.24	0.23	3.22
O13b	1.47	0.67	0.48	0.00	4.09						
O14	1.44	0.51	0.71	0.00	3.95	C14	1.43	0.77	0.34	0.00	3.92
O15	2.96	1.55	2.86	3.21	5.53	C15	2.26	1.68	0.95	0.00	4.87
Median	1.49	1.01	0.75	0.69	3.95	Median	1.77	1.01	0.95	1.16	3.79
*P*-Value	0.461	0.623	0.428	0.307	0.546						

*aa 1–226* full-length region, *aa 1–99* N-terminal region, *aa 100–169* MHR antigenic loops, *aa 124–147* “α” determinant, *aa 170–226* C-terminal region; ^a^reference sequence: genotype B (GQ205440), genotype C (GQ205441)

### Important immune escape mutations

Fragment analysis of sequences approximately 7–10 amino acids long revealed no significant differences in the amino acid substitution rate between the OBI and control patients in the majority of regions. Moreover, while the nucleotide substitution rate within amino acids 130 to 139, which belong to the MHR, in OBI patients (0.27) was significantly lower than that in the control group (0.69, *P* = 0.027, Fig. 3b), the overall percentage of amino acid substitutions within the MHR of individual patients was similar between the two groups (Table 4).

**Table 4:**
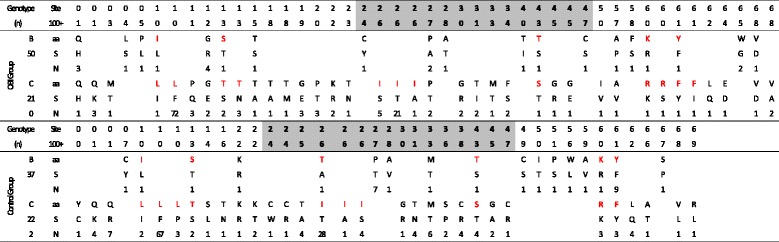
Comparison of the amino acid sequence in MHR of HBsAg between OBI group and control group

The amino acid sequence derived from each clone was compared to a reference sequence from the same genotype chosen in order to standardize our comparison. The locations of residues with regard to the MHR are given at the top of each group and gray shading region belong to “ɑ” determinant. Each reference or substituted amino acid is represented in the aa or S line, the number of clones with the change are indicated in the N line. red letter indicates HBV genotype-specific amino acid. Reference sequence: genotype B (GQ205440), genotype C (GQ205441)

Most of the sequenced clones also had a high degree of similarity to their appropriate reference sequences (genotype B or C). More than 10 % of the sequenced clones either had amino acid substitutions at s127 and s161 if it belonged to genotype B or at s110 and s126 if it belonged to genotype C, and the incidence of amino acid substitution at s110, s126, s127, and s161 in the OBI group was similar to or lower than that observed in the control group (*P* = 0.944, 0.417, 0.357, and 0.018, respectively). Substitutions at several positions, including s110, s113, s126, s143, s160, and s161, appeared to be genotype-specific (Table 4) [27, 28]. Notably, the mutations at s110, s126, and s161 have been considered high-frequency amino acid substitutions and were found in both the OBI and HBV groups, but the clinical significance of these mutations still needs to be clarified. In terms of immunogenicity, mutations typically associated with altered S protein function are known to occur at positions s123, s145, and s147. However, mutations in these positions were found in only 1, 2, and 2 clones in the OBI group, respectively.

## Discussion

Recently, several studies investigating the prevalence of OBI among blood donors from various regions of the world demonstrated that 0 to 2.87 % of the donors had OBI (reviewed by Assar et al.) [3]. The prevalence of OBI has also been found to be significantly higher in leukemia and hemodialysis patients [5, 29, 30]. Thus, the detection and analysis of OBI is essential for proper diagnosis. A few factors have been suggested to affect the detection of OBI, including the sensitivity and reliability of the technology used (e.g., the ELISA assay for detection of HBsAg vs. PCR analysis), the size of the serum sample pool, and the variation in HBV genotype in different regions/populations [31–33]. In the present study, we took various measures to address these technical issues. For example, we tested each sample using two different diagnostic kits (Kehua and Abbott) that are widely used in China. This allowed us to minimize the incidence of false negative results. Since our quantitative assay indicated that the level of HBV DNA in OBI patients was very low, we further performed nested PCRs for the S, C, and X regions using primer sets specific for these regions. By stipulating that samples must be positive for at least two of these regions to be considered HBV DNA-positive, we significantly reduced the chance for false positive results. These procedures revealed that the prevalence of OBI in families with a history of HBV infection was approximately 8.0 %, a result that is corroborated by several previous clinical investigations [4, 6, 7].

After eliminating technical bias, we sought to elucidate the underlying mechanisms involved in OBI pathogenesis, which have been suggested to be related to genetic variation, HBV reproduction levels, gene integration, suppressed expression and secretion of HBsAg, co-infection with HCV, host genetic or immune status, or any combination of these or other phenomena. Numerous studies have shown that amino acid substitutions or variations in the S gene not only occur in OBI patients, but may also be present in chronic HBV infection patients [34–36]. However, most studies in the current literature on S gene mutations in OBI patients are limited by various problems such as lack of a control group, simply using patients with chronic HBV infection in the general population as the control, small sample size, and/or retrieving sequence information simply by sequencing the PCR products without a cloning step. Thus, in order to further clarify the role of S gene genetic variation in OBI, we investigated the prevalence of OBI in Chinese families with HBV infection. The S gene from family members with chronic HBV infection or OBI was then amplified, cloned, and sequenced. In order to improve reliability and comparability, HBsAg-positive patients from families with OBI were selected as controls because they were likely to share the same source of infection as the OBI patients in addition to having similar living environments and genetic background, enabling us to provide a more meaningful comparison between the OBI group and the control group. These OBI patients and their corresponding family members in the control group could be parent–child or sibling relationships, but the differences in age and gender between the two groups were minimal. Our analysis showed that, most clones from 15 OBI patients were highly homologous to those from their HBV-infected family members, indicating that it is likely that most OBI patients share the same sources of infection with their control family members (data not shown).

Consistent with previous studies, the levels of HBV DNA detected in all of the OBI patients were lower than 1000 copies/ml, whereas some of them were negative for all other serological markers and antibodies. Some patients were also found to have abnormal liver function. Further, eight patients in both the OBI and control groups were infected with a medley of HBV genotypes, including B and C variants. Sequencing of the PCR products was performed twice for each sample in order to exclude potential single-sample contamination. Double peaks present at some genotype-specific sites suggest the existence of co-infection by different genotypes. Although mixed HBV infection was more common in the OBI group than in the control group, this difference was not statistically significant, indicating that mixed genotype HBV infection does not play a major role in OBI pathogenesis.

In this study, reliability in our methodology and results was also enhanced by using appropriate reference strains. In most previous studies investigating S gene variation, researchers utilized reference strains that did not necessarily correspond to the local strains in their respective areas [34, 35]. Instead, they used sequences that had obvious differences from the local HBV genotype reference strain, an error that we believe likely resulted in an increase in the detected number of amino acid substitutions in both OBI- and HBsAg-positive patients. This would likely affect the subsequent statistical analysis and could potentially generate bias in the analysis. Therefore, in the present study we chose the genotype B and genotype C reference strains from China, which largely decreased the number of amino acid substitutions detected in the S gene in both groups.

Furthermore, it is likely that the presence of stop codon mutations as well as deletion and insertion mutations in both groups can affect expression, secretion, and antigenicity of the HBsAg. In order to investigate the biological significance of these mutations, we cloned and sequenced the S gene from patients in both groups. Notably, stop codon mutations and deletion/insertion mutations were more common in the OBI group (13.6 % and 4.4 %, respectively) than in the control group (1.5 % and 1.9 %, respectively). However, these mutations were found in only 18.0 % of the clones sequenced for the OBI group and existed as the dominant variations in only 3 of the OBI patients investigated. Therefore, we suspect that these mutations only affect HBsAg expression, secretion, and/or antigenicity in a minority of OBI patients.

For the clones in both groups without any TID mutations, we performed a sequential sequence analysis for every 9–10 amino acids of the S gene. Our data indicated that the frequency of amino acid changes in residues 1–9, 180–189, and 220–226 of HBsAg was higher than other regions (more than 4 per 100 amino acids) in both groups, and there were no statistically significant differences between the OBI patients and the control patients for the majority of the gene. However, compared to the controls, residues 170–179 in the OBI group were more highly mutated than those in the control patients. It is also worth noting that mutations in these regions have not been previously associated with OBI pathogenesis. Alternatively, the frequencies of amino acid changes in the N-terminal, C-terminal, and MHR, including the “α” determinant area, of the S gene in the OBI group were not significantly different from those in the control group. In fact, single point mutations in the MHR and “α” determinant that could affect the antigenicity of HBsAg, such as G145R, Q129N, D144A, and T123N, were rarely found in our sequenced clones from either group, indicating that point mutations in the HBsAg S gene may play little, if any, role in OBI.

Although the low viral load of the OBI patients included in this study made it difficult to carry out whole genome cloning to fully investigate the role of mutations present in regions outside the S gene [37], we believe additional study using similar procedures will help advance our understanding of OBI. For example, the commercial Abbott kit was able to detect some mutations within the S gene, such as G145R, etc., and it may be a useful tool to exclude some “occult HBV infection” patients with G145R mutation in this study [38, 39]. Using this and similar tools as well as proper controls and reference sequences, we hope to elucidate the mechanisms responsible for HBsAg negativity in OBI patients as a better understanding of OBI will ultimately lead to more accurate diagnosis of and better treatment for chronic HBV infection.

## Conclusions

In summary, cloning of the S gene in OBI patients and their HBsAg-positive family members revealed that the total number of amino acid substitutions or variations in the S protein was not significantly altered in the majority of clones isolated from OBI patients, indicating that variations in S protein may not play a major role in OBI pathogenesis.

## Methods

### Patients and sera

From March 2010 to July 2014, we collected 747 HBsAg-negative sera samples from 265 families in which at least one family member had a history of chronic HBV infection. These samples were collected at 14 different hospitals in China. A total of 17 cases of occult HBV infection (OBI group) and 15 cases of chronic HBV infection (control group) from the same families were included in this study. The study protocol was reviewed and approved by the Ethics Committee of the First Affiliated Hospital, Anhui Medical University (PJ 20-14-05-12), and informed consent was obtained from each patient prior to inclusion in the study.

The blood samples collected from each patient were centrifuged at 950 × g for 5 min. The isolated serum from each sample was used for serological tests within 4 h. The rest of the samples were then stored at −80 °C.

### Detection of HBV serological markers

HBsAg, anti-HBs, HBeAg, anti-HBe, and anti-HBc were detected with an enzyme-linked immunosorbant assay (ELISA) using a diagnostic kit (Kehua Biotechnology, Shanghai, China). Sera samples from patients with OBI were retested for the presence of HBsAg and anti-HBs by using a standard commercially available microparticle enzyme immunoassay (AxSYM assay; Abbott, Wiesbaden, Germany) in order to confirm the absence of HBsAg in their sera. Patients were also tested for HIV and HCV using ELISA assays with diagnostic kits (Kehua Biotechnology, Shanghai, China). Serum HBV-DNA levels were measured using a TaqMan real-time PCR assay (Zhijiang Biotechnology, Shanghai, China) with a lower limit of detection of 1000 copies/ml.

### Extraction and amplification of the HBV genome

DNA was extracted from 200 μl of each patient’s serum by using a phenol-chloroform extraction method, and suspended in 20 μl of water. Nested PCR was performed using primers specific for the S, C, and X regions of the HBV genome by following the methods described by Zhang et al. [40, 41], and the detection sensitivity ranged from 20 to 50 copies/ml.

Nested PCR was also performed using specific primers targeting the full length small S gene. The primers were designed according to previously reported reference sequences (GenBank: GQ205440, GQ205441) for strains found in Chinese patients [27]. The primer sequences used in this experiment were: 5’-CTGGTGGCTCCAGTTC-3’ and 5’-CTT(G/A)TAAGTTGGCGAG-3’ (for the first round of PCR), 5’-ACGGAATTCG(T/C)ACCGAA(C/T)ATGGAGA(A/G)-3’ and 5’-GCACTGCAGTTAAATGTAT(A/G)CCCAAAG-3’ (for the second round). The first round of PCR was carried out in a final volume of 25 μl containing 2.5 μl of 10× PCR buffer which contained 15 mM MgCl_2_, 1 μl of 10 mM dNTP, 1 μl of each primer (10 mM), 2.5 units of Taq polymerase (Takara, Dalian, China), and 5 μl of DNA template. The cycling conditions were initiated with denaturation at 94 °C for 3 min, followed by 30 cycles of 94 °C for 45 s, 55 °C for 60 s, and 72 °C for 90 s. The second round of PCR was carried out using 5 μl of the PCR product obtained in the first round and 1 μl of each primer (10 mM). The cycling conditions were initiated with denaturation at 94 °C for 3 min, followed by 30 cycles of 94 °C for 45 s, 57 °C for 60 s, and 72 °C for 90 s. A final extension step was performed for 7 min at 72 °C. The amplification products (10 μl of each) were analyzed by electrophoresis on a 3.5 % agarose gel stained with ethidium bromide and observed under ultraviolet light with an Image analysis system (Tanon, Shanghai, China). The detection sensitivity was determined to be 50 copies/ml by using control plasmid pHBV1.3, which contains the full-length HBV genome. The size of the PCR product was calculated using the Tanon software (Tanon, Shanghai, China) to analyze the migration of PCR product and the DL2000 DNA marker (Takara, Dalian, China) on the agarose gel. In doing so, it appears that the product was approximately 700 bps.

### Cloning and sequencing of the S gene

Following PCR amplification and electrophoresis, the small S gene PCR products were recovered from the agarose gel, purified with the QIAquick Gel Extraction Kit (QIAGEN, Dusseldorf, Germany), and cloned using a pMD18-T cloning kit (Takara, Dalian, China). The recombinant plasmids were then isolated with a DNA extraction kit (Takara, Dalian, China), and clonal sequencing (Sangon, Shanghai, China) was performed using an automated DNA sequencer (ABI3730). Alignment and multiple comparisons of the resulting HBV sequences to two GenBank reference sequences (genotype B: GQ205440 and genotype C: GQ205441) were performed using Primer5.0 software (http://www.premierbiosoft.com/primerdesign/index.html) [27]. Genotyping of HBV was then performed by phylogenetically analyzing the S gene using sequences from the database [42].

### Statistical analysis

Statistical analyses were performed using the SPSS statistics package version 17.0 (SPSS Inc., Chicago, USA). The rate comparison was made using the chi-square criterion. Student’s *t*-test and nonparametric test were used to compare difference between groups, where appropriate. Differences corresponding to a *P* < 0.05 were considered statistically significant in all comparisons.

## Abbreviations

aa: Amino acid
ALT: Alanine aminotransferase
anti-HBc: Antibodies anti HBV Core protein
anti-HBe: Antibodies anti HBV e protein
anti-HBs: Antibodies anti HBV surface protein
ASC: asymptomatic HBV carrier
CHB: chronic hepatitis B
HBeAg: hepatitis B e antigen
HBsAg: hepatitis B surface antigen
HBV: hepatitis B virus
HCC: hepatocellular carcinoma
HCV: hepatitis C virus
LC: liver cirrhosis
MHR: major hydrophilic region
OBI: occult HBV Infection
ORF: open reading frame
PCR: polymerase chain reaction
SPSS: statistical product and service solutions
TID: termination insertion, or deletion
U: Unit
ULN: upper limit of normal
